# Prevalence, identification of virulence factors, O-serogroups and antibiotic resistance properties of Shiga-toxin producing *Escherichia coli* strains isolated from raw milk and traditional dairy products

**DOI:** 10.1186/s13756-018-0345-x

**Published:** 2018-04-16

**Authors:** Reza Ranjbar, Farhad Safarpoor Dehkordi, Mohammad Hossein Sakhaei Shahreza, Ebrahim Rahimi

**Affiliations:** 1 Molecular Biology Research Center, Systems Biology and Poisonings Institute, Baqiyatallah University of Medical Sciences, Tehran, Iran; 2 Young Researchers and Elites Club, Shahrekord Branch, Islamic Azad University, Shahrekord, Iran; 3 Doctor Veterinary Medicine, Faculty of Veterinary Medicine, Shahrekord Branch, Islamic Azad University, Shahrekord, Iran; 4 Department of Food Hygiene and Public health, Faculty of Veterinary Medicine, Shahrekord Branch, Shahrekord, Iran

**Keywords:** Shiga toxin producing *Escherichia coli*, Molecular characterization, Antibiotic resistance

## Abstract

**Background:**

Shiga-toxigenic *Escherichia coli* strains are one of the most important foodborne bacteria with an emergence of antibiotic resistance. Foodborne STEC strains are mainly associated with presence of certain virulence factors and O-seogroups. The present investigation was done to study the distribution of virulence factors, O-serogroups and antibiotic resistance properties of Shiga-toxigenic *Escherichia coli* isolated from milk and dairy products.

**Methods:**

Six-hundred samples were randomly collected and immediately transferred to laboratory. All samples were cultured and *E. coli* strains were isolated. STEC strains were identified based on the presence of putative virulence factors and subtypes. STEC isolates were subjected to multiplex PCR and disk diffusion methods.

**Results:**

One-hundred and eighty-one out of 600 samples (30.16%) harbored *E. coli*. Prevalence of STEC strains was 10.66%. O157 (43.75%) and O26 (37.50%) were the most frequently identified serogroups. *Aac(3)-IV* (100%), *CITM* (96.87%) and *tetA* (76.56%) were the most commonly detected antibiotic resistance genes. STEC strains had the highest prevalence of resistance against ampicillin (100%), gentamicin (100%) and tetracycline (96.87%).

**Conclusions:**

Kashk and dough were negative for presence of *E. coli* strains. High prevalence of resistant-O157 strains and simultaneous presence of multiple virulence factors pose an important public health problem regarding the consumption of raw milk and dairy products.

## Background

Billions of people consuming milk and dairy products every day. Milk and dairy products are full from valuable nutritional factors such as proteins, lipids, minerals, and vitamins. Researches showed that consumption of milk and dairy products is useful for human [1, 2]. There are several types of dairy products such as cheese, cream, butter, yoghurt, and ice cream, with high beneficial effects for human health [3, 4]. Kashk and dough are two important traditional dairy products in Iran and some other sites of the world. Kashk comes in liquid or dried forms and is traditionally made with the milk left over from cheese-making. Dough is a popular salty yogurt-based beverage amongst the Iranian people [3, 4]. Based on the high consumption rate of milk and dairy products, it is important to ensure from the microbial quality of these types of food samples [5].

*Escherichia coli* (*E. coli*) strains are one of the most important cause of foodborne diseases al-around the world [5–7]. Milk and dairy products are one of the main sources of transmission of the *E. coli* strains into the human [5–7]. *E. coli* is a Gram-negative, non-sporulating, flagellated, rod-shaped and facultative anaerobic bacterium which belongs to Enterobacteriaceae family. Enterohemorrhagic *E. coli* (EHEC) strains are a subtype of the Vero (Shiga) toxin (Vtx or Stx)-producing *E. coli* (VTEC or STEC) [5, 6, 8]. EHEC bacteria are causative agents of severe syndromes including Hemolytic Uremic Syndrome (HUS), Thrombotic Thrombocytopenic Purpura (TTP), Hemorrhagic Colitis (HC) and bloody and non-bloody diarrhea [5, 6, 9].

*E. coli* strains isolated from milk and dairy products harbored the high prevalence of intimin (*eaeA*), Vero or Shiga toxins (*vtx1* and *vtx2* or *stx1* and *stx2*) and hemolysin (*hlyA*). These factors can cause bacterial adhesion and invasion into the intestinal epithelial cells which cause severe attaching-effacing (A/E) lesions [9–11]. Attaching and effacing lesions are mainly caused by certain types of the STEC bacteria called Attaching-Effacing *E. coli* (AEEC) [9–11]. Expression of the *eae* gene is the main factor for occurrence of A/E lesions [9–11].

Occurrence of foodborne diseases caused by this bacterium are mainly occurred due to the activity of certain O-serogroups including non-O157 (O103, O26, O113, O91, O145, O111, O121, O128 and O45) and also O157 [5, 6, 12].

STEC strains are mainly resistant against several types of antibiotics. Documented data revealed that STEC strains isolated from milk and dairy products and also other types of food samples harbored the high prevalence of resistance against different types of antibiotics including aminoglycosides, fluoroquinolone, tetracyclines, trimethoprim, ampicillin, cephalothin, sulfonamides, gentamicin and chloramphenicol [5–13]. Molecular epidemiological researches showed that presence of certain antibiotic resistance genes including the genes that encode resistance against fluoroquinolone (*qnr*), trimethoprim (*dfrA1*), cephalothin (*blaSHV*), tetracycline (*tetA* and *tetB*), ampicillin (*CITM*), gentamicin (*aac(3)-IV*), sulfonamide (*sul1*), chloramphenicol (*cat1* and *cmlA*), aminoglycosides (*aadA1*), and erythromycin (*ereA*) is the most important reason for occurrence of antibiotic resistance in STEC strains [5–13].

According to an uncertain role of STEC strains in milk and dairy products and deficiency of similar microbiological and epidemiological surveys in Iran, the current examination was done to molecular characterization of O-serogroups, virulence genes and antibiotic resistance properties of STEC bacteria recovered raw milk and traditional dairy products.

## Methods

### Samples and *Escherichia coli* identification

From March 2015 to March 2016, a total of 600 different types of raw milk and traditional dairy product samples including raw bovine (*n* = 70), raw ovine (*n* = 60), raw caprine (n = 60), raw buffalo (*n* = 40) and raw camel (*n* = 30) milk samples and traditional cheese (*n* = 50), yoghurt (*n* = 60), kashk (*n* = 50), dough (*n* = 50), butter (*n* = 40), cream (*n* = 40) and ice-cream (*n* = 50) samples were randomly collected from Isfahan province, Iran. Samples collection was done according to the instruction introduced by International Dairy Federation [14]. Samples (100 mL) were transferred to laboratory at 4 °C. All food samples showed normal physical characters including odor, color and consolidation.

Twenty-five milliliters of raw milk samples and 25 mg of homogenized dairy products were added to sterilized tubes containing 225 ml of Buffered Peptone Water (BPW, Merck, Germany) and hatched aerobically at 37 °C for 24 h. Then, samples were cultured on 5% sheep blood and MacConkey agar (Merck, Germany) media and incubated at 37 °C for 24 h. Colonies with the typical color and appearance of *E. coli* were picked and streaked again on blood agar plates and re-streaked on EMB agar (Merck, Germany). All plates were further incubated for 24 h at 37 °C. The green metallic sheen colonies were considered as *E. coli*. The presumptive colonies were biochemically tested for growth on triple sugar iron agar (TSI) and lysine iron agar (LIA), oxidative/fermentative degradation of glucose, citrate utilization, urease production, indol fermentation, tryptophan degradation, glucose degradation (methyl red test) and motility.

### PCR confirmation of *E. coli* isolates

Colonies were further confirmed using the *16S rRNA*-based Polymerase Chain Reaction (PCR) according to the method described previously [15]. Bacterial strains were sub-cultured overnight in Luria-Bertani broth (Merck, Germany) and further incubated for 48 h at 37 °C. Genomic DNA was extracted from bacterial colonies using the DNA extraction kit (Thermo Fisher Scientific, St. Leon-Rot, Germany) according to manufacturer’s instruction. DNA quality and concentration were examined using the spectrophotometer [16]. The 10 ml bacterial DNA extract and controls were amplified with 0.5 mM primers (Forward: 5’-AGTTTGATCCTGGCTCAG-3′ and Reverse: 5’-AGGCCCGGGAACGTATTCAC-3′) (1343 bp) [15], 200 mM of each dNTP (Thermo Fisher Scientific, St. Leon-Rot, Germany), 2 mM MgCl2, 10 mM KCl PCR buffer and 1.0 U Taq polymerase (Thermo Fisher Scientific, St. Leon-Rot, Germany). The DNA was amplified in a programmable thermal cycler (Eppendorf, Mastercycler® 5330, Eppendorf-Netheler-Hinz GmbH, Hamburg, Germany) PCR device using the following protocol: 94 °C for 5 min, 40 cycles of 94 °C for 1 min, 55 °C for 1 min, 72 °C for 2 min, and final 72 °C for 5 min.

### Detection of virulence factors, O-serogroups and antibiotic resistance genes

Table 1 indicates list of primers and PCR conditions used for detection of O-serogroups, virulence genes and antimicrobial resistant genes [17, 18]. Fifteen microliters of amplified PCR products were subjected to electrophoresis in a 1.5% agarose gel in 1X TBE buffer at 80 V for 30 min, stained with SYBR Green (Thermo Fisher Scientific, St. Leon-Rot, Germany) [17, 18]. All runs included a negative DNA control consisting of PCR grade water and strains of *E. coli* O157:K88 ac:H19, CAPM 5933 and *E. coli* O159:H20, CAPM 6006 were used as positive controls.

**Table 1:** Primers and PCR conditions used for characterization of virulence genes, O-serogroups and antibiotic resistance genes in the STEC strains isolated from raw milk and traditional dairy product samples

Target gene	Primer sequence (5′-3′)	PCR product (bp)	PCR programs	PCR Volume (50 μL)
O157	F: CGGACATCCATGTGATATGG R: TTGCCTATGTACAGCTAATCC	259	1 cycle: 95 ^0C^ ------------ 3 min.	5 μL PCR buffer 10X 2 mM Mgcl_2_
O145	F: CCATCAACAGATTTAGGAGTG R: TTTCTACCGCGAATCTATC	609	30 cycle: 95 ^0C^ ------------ 20 s 58 ^0C^ ------------ 40 s 72 ^0C^ ------------ 30 s	150 μM dNTP (Fermentas) 0.75 μM of each primers F & R 1.5 U Taq DNA polymerase (Fermentas)
O103	F: TTGGAGCGTTAACTGGACCT R: GCTCCCGAGCACGTATAAG	321		
O26	F: CAGAATGGTTATGCTACTGT R: CTTACATTTGTTTTCGGCATC	423		
O111	F: TAGAGAAATTATCAAGTTAGTTCC R: ATAGTTATGAACATCTTGTTTAGC	406	1 cycle: 72 ^0C^ ------------ 8 min	3 μL DNA template
O91	F: GCTGACCTTCATGATCTGTTGA R: TAATTTAACCCGTAGAATCGCTGC	291	1 cycle: 94 ^0C^ ------------ 6 min. 34 cycle: 95 ^0C^ ------------ 50 s 58 ^0C^ ------------ 70 s 72 ^0C^ ------------ 55 s 1 cycle: 72 ^0C^ ------------ 10 min	5 μL PCR buffer 10X 2 mM Mgcl_2_ 150 μM dNTP (Fermentas) 0.75 μM of each primers F & R 1.5 U Taq DNA polymerase (Fermentas) 3 μL DNA template
O128	F: GCTTTCTGCCGATATTTGGC R: CCGACGGACTGATGCCGGTGATT	289		
O121	F: TGGCTAGTGGCATTCTGATG R: TGATACTTTAGCCGCCCTTG	322		
O113	F: GGGTTAGATGGAGCGCTATTGAGA R: AGGTCACCCTCTGAATTATGGCAG	771		
O45	F: CCGGGTTTCGATTTGTGAAGGTTG R: CACAACAGCCACTACTAGGCAGAA	527		
*stx1*	F: AAATCGCCATTCGTTGACTACTTCT R: TGCCATTCTGGCAACTCGCGATGCA	366	1 cycle: 95 ^0C^ ------------ 3 min. 34 cycle: 94 ^0C^ ------------ 60 s 56 ^0C^ ------------ 45 s 72 ^0C^ ------------ 60 s 1 cycle: 72 ^0C^ ------------ 10 min	5 μL PCR buffer 10X 2 mM Mgcl_2_ 150 μM dNTP (Fermentas) 0.75 μM of each primers F & R 1.5 U Taq DNA polymerase (Fermentas) 3 μL DNA template
*stx2*	F: CGATCGTCACTCACTGGTTTCATCA R: GGATATTCTCCCCACTCTGACACC	282		
*eaeA*	F: TGCGGCACAACAGGCGGCGA R: CGGTCGCCGCACCAGGATTC	629		
*ehly*	F: CAATGCAGATGCAGATACCG R: CAGAGATGTCGTTGCAGCAG	432		
*aadA1*	F: TATCCAGCTAAGCGCGAACT R: ATTTGCCGACTACCTTGGTC	447	1 cycle: 94 ^0C^ ------------ 8 min. 32 cycle: 95 ^0C^ ------------ 60 s 55 ^0C^ ------------ 70 s 72 ^0C^ ------------ 2 min 1 cycle: 72 ^0C^ ------------ 8 min	5 μL PCR buffer 10X 2 mM Mgcl_2_ 150 μM dNTP (Fermentas) 0.75 μM of each primers F & R 1.5 U Taq DNA polymerase (Fermentas) 3 μL DNA template
*tetA*	F: GGTTCACTCGAACGACGTCA R: CTGTCCGACAAGTTGCATGA	577		
*tetB*	F: CCTCAGCTTCTCAACGCGTG R: GCACCTTGCTGATGACTCTT	634		
*dfrA1*	F: GGAGTGCCAAAGGTGAACAGC R: GAGGCGAAGTCTTGGGTAAAAAC	367		
*qnr*	F: GGGTATGGATATTATTGATAAAG R: CTAATCCGGCAGCACTATTTA	670		
*aac(3)-IV*	F: CTTCAGGATGGCAAGTTGGT R: TCATCTCGTTCTCCGCTCAT	286		
*sul1*	F: TTCGGCATTCTGAATCTCAC R: ATGATCTAACCCTCGGTCTC	822		
*blaSHV*	F: TCGCCTGTGTATTATCTCCC R: CGCAGATAAATCACCACAATG	768		
*CITM*	F: TGGCCAGAACTGACAGGCAAA R: TTTCTCCTGAACGTGGCTGGC	462		
*cat1*	F: AGTTGCTCAATGTACCTATAACC R: TTGTAATTCATTAAGCATTCTGCC	547		
*cmlA*	F: CCGCCACGGTGTTGTTGTTATC R: CACCTTGCCTGCCCATCATTAG	698		

### Disk diffusion test

Antibiotic resistance pattern of *E. coli* isolates was determined by simple disk diffusion method. The Mueller–Hinton agar (Merck, Germany) media were used for antibiotic susceptibility test. Principles of the Clinical and Laboratory Standards Institute (CLSI) were used for this purpose [19]. Susceptibility of *E. coli* isolates were tested against tetracycline (30 u/disk), ampicillin (10 u/disk), cefotaxime (30 μg/disk), gentamicin (10 μg/disk), ciprofloxacin (5 μg/disk), amikacin (30 u/disk), ceftazidime (30 μg/disk), imipenem (30 u/disk), cotrimoxazole (30 μg/disk), enrofloxacin (5 μg/disk), sulfamethoxazole (25 μg/disk), trimethoprim (5 μg/disk), streptomycin (10 μg/disk) and chloramphenicol (30 μg/disk) antibiotic agents (Oxoid, UK). All of the inoculated plates were aerobically incubated at 37 °C for 18-24 h. Results were interpreted based on the instruction provided by CLSI [19]. *E. coli* ATCC 25922 was used as positive control.

### Statistical analysis

Data obtained from all tests were transferred to the Microsoft Excel spreadsheet (Microsoft Corp., Redmond, WA) for analysis. At first, all data were subjected to Kolmogorov-Smirnov test to study their distribution. Then, the statistical analysis was performed using SPSS/20.0 software (SPSS Inc., Chicago, IL). *P*-values were calculated using Chi-square and Fisher’s exact tests. Statistical analysis was used to find any significant relationship for prevalence of *E. coli* and also STEC strains between different samples, seasonal distribution of bacteria, distribution of O-serogroups, virulence factors and antibiotic resistance. The *P*-value less than 0.05 was considered statistically significant.

## Results

Table 2 represents the prevalence of *E. coli* strains in different types of milk samples and dairy products. One-hundred and eighty-one out of 600 samples (30.16%) were positive for *E. coli* strains. Cheese (80%) and raw buffalo milk (50%) samples had the highest prevalence of *E. coli*, while raw camel milk (6.66%) had the lowest. There were no positive results for Kashk and dough samples. Statistical significant difference was seen between types of samples and prevalence of *E. coli* (*P* < 0.05).

**Table 2:** Prevalence of *Escherichia coli* strains isolated from raw milk and traditional dairy product samples

Types of samples	No. samples collected	No positive strains (%)
Raw bovine milk	70	30 (42.85)
Raw ovine milk	60	21 (35)
Raw caprine milk	60	23 (38.33)
Raw buffalo milk	40	20 (50)
Raw camel milk	30	2 (6.66)
Cheese	50	40 (80)
Yoghurt	60	5 (8.33)
Kashk	50	–
Dough	50	–
Butter	40	10 (25)
Cream	40	15 (37.50)
Ice-cream	50	15 (30)
Total	600	181 (30.16)

**Fig. 1:**
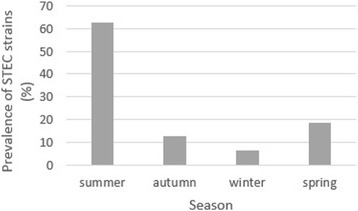
Seasonal prevalence of *Escherichia coli* strains in raw milk and traditional dairy product samples

Figure 1 revealed the seasonal prevalence of *E. coli* strains. Raw milk and traditional dairy product samples which were collected through the summer season had the highest prevalence of *E. coli* (62.50%). Statistical significant difference was seen for the prevalence of *E. coli* strains between hot and cold seasons (*P* = 0.018).

Table 3 represents the prevalence of EHEC and AEEC subtypes in the *E. coli* strains isolated from raw milk and traditional dairy product samples. Prevalence of EHEC subtype in raw bovine, ovine, caprine and buffalo milk and traditional cheese, yoghurt, butter, cream and ice-cream samples was 25%, 22.22%, 27.27%, 35.71%, 32%, 33.33%, 16.66%, 25% and 22.22%, respectively. Prevalence of AEEC subtype in raw bovine, ovine, caprine and buffalo milk and traditional cheese, yoghurt, butter, cream and ice-cream samples was 31.25%, 33.33%, 36.36%, 35.71%, 36%, 33.33%, 50%, 50% and 55.55%, respectively. *E. coli* strains of the EHEC subtype were positive for all *stx1*, *eae* and *ehly* virulence genes. Statistical significant difference was seen between the prevalence of EHEC and AEEC subtypes (*P* < 0.01). Sixty-four out of 181 *E. coli* isolates (35.35%) were positive for AEEC and EHEC subtypes. These isolates were determined as STEC strains. There were no STEC strains in the raw camel milk samples. *E. coli* strains of the EHEC subtype were recognized as O157 serogroup.

**Table 3:** Prevalence of pathogenic subtypes and related virulence factors in *Escherichia coli* strains isolated from raw milk and traditional dairy product samples

Samples (No. positive)	Subtypes	No. positive samples	Virulence genes
Raw bovine milk (30)	Non detected	7 (43.75)	–
	EHEC	4 (25)	*stx1*, *eae*, *ehly*: 4 (100)
	AEEC	5 (31.25)	*stx1*: 5 (100) *stx2*: 3 (60) *eaeA*: 5 (100) *stx1, eaeA*: 3 (60) *stx2, eaeA*: 2 (40) *stx1, stx2, eaeA*: 1 (20)
	Total	16 (53.33)	
Raw ovine milk (21)	Non detected	4 (44.44)	–
	EHEC	2 (22.22)	*stx1*, *eae*, *ehly*: 2 (100)
	AEEC	3 (33.33)	*stx1*: 3 (100) *stx2*: 1 (33.33) *eaeA*: 3 (100) *stx1, eaeA*: 1 (33.33) *stx2, eaeA*: 1 (33.33) *stx1, stx2, eaeA*: 1 (33.33)
	Total	9 (42.85)	
Raw caprine milk (23)	Non detected	4 (36.36)	–
	EHEC	3 (27.27)	*stx1*, *eae*, *ehly*: 2 (100)
	AEEC	4 (36.36)	*stx1*: 4 (100) *stx2*: 2 (50) *eaeA*: 4 (100) *stx1, eaeA*: 2 (50) *stx2, eaeA*: 1 (25) *stx1, stx2, eaeA*: 1 (25)
	Total	11 (47.82)	
Raw buffalo milk (20)	Non detected	4 (28.57)	–
	EHEC	5 (35.71)	*stx1*, *eae*, *ehly*: 5 (100)
	AEEC	5 (35.71)	*stx1*: 5 (100) *stx2*: 3 (60) *eaeA*: 5 (100) *stx1, eaeA*: 3 (60) *stx2, eaeA*: 1 (20) *stx1, stx2, eaeA*: 1 (20)
	Total	14 (70)	
Raw camel milk (2)	Non detected	1 (100)	–
	EHEC	–	*–*
	AEEC	–	*stx1*: *stx2*: - *eaeA*: *stx1, eaeA*: *stx2, eaeA*: - *stx1, stx2, eaeA*: -
	Total	1 (50)	
Cheese (40)	Non detected	8 (32)	–
	EHEC	8 (32)	*stx1*, *eae*, *ehly*: 8 (100)
	AEEC	9 (36)	*stx1*: 9 (100) *stx2*: 5 (55.55) *eaeA*: 8 (88.88) *stx1, eaeA*: 5 (66.66) *stx2, eaeA*: 2 (22.22) *stx1, stx2, eaeA*: 2 (22.22)
	Total	25 (62.50)	
Yoghurt (5)	Non detected	1 (33.33)	–
	EHEC	1 (33.33)	*stx1*, *eae*, *ehly*: 1 (100)
	AEEC	1 (33.33)	*stx1*: 1 (100) *stx2*: 1 (100) *eaeA*: 1 (100) *stx1, eaeA*: 1 (100) *stx2, eaeA*: 1 (100) *stx1, stx2, eaeA*: 1 (100)
	Total	3 (60)	
Butter (10)	Non detected	2 (33.33)	–
	EHEC	1 (16.66)	*stx1*, *eae*, *ehly*: 1 (100)
	AEEC	3 (50)	*stx1*: 3 (100) *stx2*: 1 (33.33) *eaeA*: 3 (100) *stx1, eaeA*: 1 (33.33) *stx2, eaeA*: 1 (33.33) *stx1, stx2, eaeA*: 1 (33.33)
	Total	6 (60)	
Cream (15)	Non detected	2 (25)	–
	EHEC	2 (25)	*stx1*, *eae*, *ehly*: 2 (100)
	AEEC	4 (50)	*stx1*: 4 (100) *stx2*: 2 (50) *eaeA*: 3 (75) *stx1, eaeA*: 2 (50) *stx2, eaeA*: 1 (25) *stx1, stx2, eaeA*: 1 (25)
	Total	8 (53.33)	
Ice-cream (15)	Non detected	2 (22.22)	–
	EHEC	2 (22.22)	*stx1*, *eae*, *ehly*: 2 (100)
	AEEC	5 (55.55)	*stx1*: 5 (100) *stx2*: 3 (60) *eaeA*: 5 (100) *stx1, eaeA*: 2 (40) *stx2, eaeA*: 2 (40) *stx1, stx2, eaeA*: 1 (20)
	Total	9 (60)	

Table 4 represents the prevalence of O157 and non-O157 serogroups in the STEC strains isolated from raw milk and traditional dairy product samples. O157 (43.75%) and O26 (37.50%) were the most commonly detected O-serogroups. Prevalence of O111, O45, O113 and O121 serogroups were 9.37%, 1.50%, 1.50% and 6.25%, respectively. There were no positive results for O145, O91 and O128 serogroups. Statistical significant difference was seen between types of samples and prevalence of O-serogroups (*P* < 0.05).

**Table 4:** Prevalence of O-serogroups in the STEC strains isolated from raw milk and traditional dairy product samples

Samples (No. STEC strains)	Distribution of O-serogroups (%)									
	O157	O26	O103	O111	O145	O45	O91	O113	O121	O128
Raw bovine milk (9)	4 (44.44)	3 (33.33)	–	1 (11.11)	–	–	–	–	1 (11.11)	–
Raw ovine milk (5)	2 (40)	2 (40)	–	1 (20)	–	–	–	–	–	–
Raw caprine milk (7)	3 (42.85)	2 (28.57)	–	–	–	1 (14.28)	–	–	1 (14.28)	–
Raw buffalo milk (10)	5 (50)	4 (40)	–	1 (10)	–	–	–	–	–	–
Cheese (17)	8 (47.05)	6 (35.29)	–	1 (5.88)	–	–	–	1 (5.88)	1 (5.88)	–
Yoghurt (2)	1 (50)	1 (50)	–	–	–	–	–	–	–	–
Butter (4)	1 (25)	2 (50)	–	1 (25)	–	–	–	–	–	–
Cream (6)	2 (33.33)	2 (33.33)	–	1 (16.66)	–	–	–	–	1 (16.66)	–
Ice-cream (4)	2 (50)	2 (50)	–	–	–	–	–	–	–	–
Total (64)	28 (43.75)	24 (37.50)	–	6 (9.37)	–	1 (1.56)	–	1 (1.56)	4 (6.25)	–

Table 5 shows the distribution of antibiotic resistance genes amongest the STEC strains isolated from raw milk and traditional dairy product samples. *Aac(3)-IV* (100%), *CITM* (96.87%) and *tetA* (76.56%) were the most commonly detected antibiotic resistance genes. Prevalence of *cmlA* (1.50%), *cat1* (18.75%) and *tetB* (20.31%) antibiotic resistance genes were low. Statistical significant difference was seen between the types of samples and distribution of antibiotic resistance genes (*P* < 0.05).

**Table 5:** Prevalence of antibiotic resistance genes in the STEC strains isolated from raw milk and traditional dairy product samples

Samples (No. STEC strains)	Distribution of antibiotic resistance genes (%)										
	*aadA1*	*tetA*	*tetB*	*dfrA1*	*qnr*	*aac(3)-IV*	*sul1*	*blaSHV*	*CITM*	*cat1*	*cmlA*
Raw bovine milk (9)	3 (33.33)	7 (77.77)	1 (11.11)	5 (55.55)	1 (11.11)	9 (100)	4 (44.44)	3 (33.33)	9 (100)	1 (11.11)	–
Raw ovine milk (5)	3 (60)	4 (80)	1 (20)	3 (60)	1 (20)	5 (100)	2 (40)	1 (20)	5 (100)	1 (20)	–
Raw caprine milk (7)	4 (57.14)	5 (71.42)	2 (28.57)	4 (57.14)	2 (28.57)	7 (100)	2 (28.57)	2 (28.57)	7 (100)	1 (14.28)	–
Raw buffalo milk (10)	3 (30)	8 (80)	2 (20)	6 (60)	3 (30)	10 (100)	5 (50)	3 (30)	9 (90)	2 (20)	–
Cheese (17)	7 (41.17)	12 (70.58)	4 (23.52)	8 (47.05)	4 (23.52)	17 (100)	7 (41.17)	5 (29.41)	16 (94.11)	4 (23.52)	1 (755.88
Yoghurt (2)	1 (50)	2 (100)	–	1 (50)	–	2 (100)	1 (50)	1 (50)	2 (100)	–	–
Butter (4)	3 (75)	3 (75)	1 (25)	2 (50)	1 (25)	4 (100)	2 (50)	1 (25)	4 (100)	1 (25)	–
Cream (6)	4 (66.66)	5 (83.33)	1 (16.66)	3 (50)	1 (16.66)	6 (100)	2 (33.33)	2 (33.33)	6 (100)	1 (16.66)	–
Ice-cream (4)	3 (75)	3 (75)	1 (25)	3 (75)	1 (25)	4 (100)	2 (50)	2 (50)	4 (100)	1 (25)	–
Total (64)	31 (48.43)	49 (76.56)	13 (20.31)	35 (54.68)	14 (21.87)	64 (100)	27 (42.18)	20 (31.25)	62 (96.87)	12 (18.75)	1 (1.56)

Table 6 indicates the prevalence of antibiotic resistance in the STEC strains isolated from raw milk and traditional dairy product samples. STEC strains harbored the highest prevalence of resistance against ampicillin (100%), gentamicin (100%) and tetracycline (96.87%) antibiotics. Statistical significant difference was seen between the types of samples and prevalence of antibiotic resistance (*P* < 0.05).

**Table 6:** Pattern of antibiotic resistance for STEC strains isolated from raw milk and traditional dairy product samples

Antibiotic resistance	Samples (No. STEC strains)									
	Raw bovine milk (9)	Raw ovine milk (5)	Raw caprine milk (7)	Raw buffalo milk (10)	Cheese (17)	Yoghurt (2)	Butter (4)	Cream (6)	Ice-cream (4)	Total (64)
Tetracycline	9 (100)	5 (100)	7 (100)	9 (90)	16 (94.11)	2 (100)	4 (100)	6 (100)	4 (100)	62 (96.87)
Ampicillin	9 (100)	5 (100)	7 (100)	10 (100)	17 (100)	2 (100)	4 (100)	6 (100)	4 (100)	64 (100)
Gentamicin	9 (100)	5 (100)	7 (100)	10 (100)	17 (100)	2 (100)	4 (100)	6 (100)	4 (100)	64 (100)
Amikacin	6 (66.66)	3 (60)	5 (71.42)	5 (50)	12 (70.58)	1 (50)	3 (75)	3 (50)	2 (50)	40 (62.50)
Imipenem	1 (11.11)	–	–	1 (10)	2 (11.76)	–	–	–	–	4 (6.25)
Sulfamethoxazole	5 (55.55)	3 (60)	2 (28.57)	5 (50)	7 (41.17)	1 (50)	2 (50)	3 (50)	2 (50)	30 (46.87)
Cefotaxime	4 (44.44)	2 (40)	1 (14.28)	3 (30)	3 (41.17)	–	1 (25)	1 (16.66)	1 (25)	16 (25)
Ciprofloxacin	5 (55.55)	3 (60)	4 (57.14)	4 (40)	12 (70.58)	1 (50)	3 (75)	3 (50)	2 (50)	37 (57.81)
Enrofloxacin	5 (55.55)	3 (60)	5 (42.85)	4 (40)	13 (76.47)	1 (50)	3 (75)	3 (50)	3 (75)	40 (62.50)
Cotrimoxazole	4 (44.44)	3 (60)	2 (28.57)	3 (30)	6 (35.29)	–	2 (50)	2 (33.33)	2 (50)	24 (37.50)
Ceftazidime	4 (44.44)	2 (40)	1 (14.28)	3 (30)	4 (23.52)	–	2 (50)	2 (33.33)	1 (25)	19 (29.68)
Trimethoprim	4 (44.44)	3 (60)	2 (28.57)	5 (50)	8 (47.05)	1 (50)	2 (50)	3 (50)	2 (50)	30 (46.87)
Streptomycin	4 (44.44)	3 (60)	4 (57.14)	4 (40)	8 (47.05)	–	2 (50)	2 (33.33)	1 (25)	29 (45.31)
Chloramphenicol	2 (22.22)	1 (20)	–	2 (20)	–	–	–	1 (16.66)	1 (25)	3 (4.68)

**Fig. 2:**
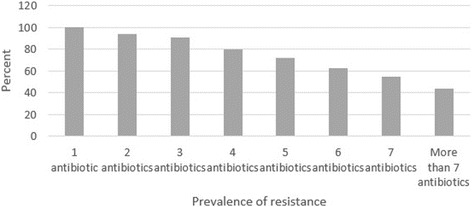
Prevalence of multi-drug resistant STEC bacteria isolated from raw milk and traditional dairy product samples

Figure 2 shows the prevalence of multi-drug resistant STEC strains isolated from raw milk and traditional dairy product samples. All of the STEC strains were resistant to at least an antibiotic, while prevalence of resistance against more than 7 antibiotics was 43.75%.

## Discussion

As far as we know, the current research is the most comprehensive report of the molecular characterization and study the phenotypic and genotypic properties of antibiotic resistance of the STEC strains isolated from raw milk and traditional dairy product samples. We found that 30.16% of raw milk and traditional dairy product samples were contaminated with *E. coli* strains. We also found that the prevalence of STEC strains were 35.35% (64/181). Ombarak et al. (2016) [20] reported that the prevalence of *E. coli* strains in various types of dairy products had a range of 21 to 77%. Elhadidy and Mohammed (2012) [21] revealed that 11.29% of cheese samples were contaminated with the STEC strains which was lower than our findings (34%). Total prevalence of *E. coli* strains in raw milk and traditional dairy product samples of studies conducted on Switzerland [22], Iran [23], Italy [24], Egypt [25], Turkey [26], China [27], and Spain [28] had range between 1% to 27% which were lower than our reported prevalence rate.

There were some probable explanations for the high prevalence of *E. coli* and also STEC strains in raw milk and traditional dairy product samples of our study [5, 7, 29]. At first. Iranian ranchers used from their hand and also traditional equipment for milking procedure which increase the risk of transmission of bacteria into the milk and dairy products [5, 7, 29]. At second, lack of maintenance of raw milk samples at temperatures below than 4 °C which facilitates survival and proliferation of bacteria [5, 7, 29]. At third, using from unpasteurized milk for production of dairy products which facilitates the survival of bacteria in dairy products. [5, 7, 29]. Transmission of pathogenic agents from the infected staffs of the milking halls and also factories is another important risk factor. There were no positive results for kashk and dough samples. Production of these two dairy products need high thermal processing which caused to destruction of pathogenic agents.

We found marked seasonality in the distribution of *E. coli* strains in raw milk and traditional dairy product samples. High prevalence of *E. coli* strains in summer season (64.10%) could be related to the low levels of individual hygiene in this season. The higher prevalence of STEC strains may be due to the higher activity of bacteria in hot seasons. Of studies which have been conducted in this field [29–31], all have shown a seasonal distribution for *E. coli* strains with a higher prevalence of bacteria in warmer seasons [32–34].

Results of the current research revealed that raw milk and traditional dairy product samples harbored the high prevalence of virulent STEC strains. We found that STEC strains harbored the high prevalence of *stx1*, *stx2*, *eaeA* and *ehlyA* virulence factors. Some isolates were positive for multiple virulence genes which represented their high pathogenicity. Momtaz et al. [5] revealed that *stx1*, *papA*, *cnf1*, *traT*, *cnf2*, *eaeA and ehly* were the most commonly detected virulence factors amongst the STEC *strains of* dairy products*.* Dehkordi et al. [7] reported that the prevalence of stx1, stx2, eaeA and ehly genes amongst the STEC strains of fermented dairy products were 44%, 30%, 36% and 28%, respectively. We found that AEEC subtypes had the higher prevalence than EHEC subtype. This finding had also been reported by other researchers [5–7, 17, 18, 28, 29]. All of the previously published articles in this field [5–7, 17, 18, 28, 29] found that EHEC strains harbored all stx1, eae and ehly genes together which was similar to our findings. Simultaneous presence of stx1 and eaeA and also stx1 and ehly virulence factors in the STEC strains is another important finding of the present study which was supported by previous researches [5–7, 17, 18, 28, 29]*.*

We found that both O157 and non-O157 (especially O26, O111 and O121) serogroups had a considerable prevalence in *STEC strains isolated from* raw milk and traditional dairy product samples. These serogroups were also predominant in the STEC strains isolated from ground beef (Argentina) [30], hospital foods (Iran) [31], dairy products (France) [32] and milk (Spain) [33]. Hemmatinezhad et al. (2015) [34] reported that the prevalence of O103, O121 and O128 serogroups in STEC strains isolated from poultry meat samples were 3.84%, 11.53% and 5.76%, respectively. Their findings revealed that O26 (30.76%) and O157 (25.96%) were the most commonly detected O-serogroups. High prevalence of O157 serogroup in the STEC strains of our research and also those of other investigations showed that collected samples were contaminated with animal sources and especially feces. Previous studies which were conducted in Turkey [35], Canada [36] and Africa [37] had similar conclusions.

Majority of the STEC strains of our research harbored the high prevalence of resistance against commonly used antibiotics. We found that phenotypic resistance pattern of STEC strains was supported by the genotypic resistance pattern. In the other hand, high prevalence of resistance of STEC strains isolated from raw milk and traditional dairy product samples was accompanied with high prevalence of antibiotic resistance genes. Multi-drug resistance was also seen for some of the STEC strains isolated from raw milk and traditional dairy product samples. Illegal and inaccurate prescription of antibiotics especially in veterinary medicine is may be the main reason for the high prevalence of antibiotic resistance in the STEC strains isolated from raw milk and traditional dairy product samples. Dehkordi et al. (2014) [7] revealed that the STEC strains isolated from fermented dairy products showed a high prevalence of resistance against tetracycline (84%), penicillin (46%), cephalothin (42%), ampicillin (38%) and streptomycin (36%). They showed that high prevalence of resistance against commonly used antibiotics was also accompanied with high prevalence of the genes that encode resistance to tetracycline (*tetA* (76%) and *tetB* (70%)), cephalothin (*blaSHV* (38%)), ampicillin (*CITM* (36%)) and gentamicin (*aac(3)-IV* (32%)). Previous study [31] showed that the prevalence of *aac(3)-IV*, *CITM*, *tetA*, *dfrA1* and *sul1* antibiotic resistance genes amongst the STEC strains isolated from food samples were 100%, 100%, 62.50%, 56.25% and 56.25%, respectively. Tark et al. (2017) [38] determined that *E. coli* strains isolated from raw milk samples harbored the high prevalence of resistance against tetracycline (23.3%), streptomycin (17.10%), ampicillin (16.60%), neomycin (11.80%), and trimethoprim/sulfamethoxazole (11.20%). Fifteen percent of their isolates had resistance against more than three classes of antibiotics. Study in Ethiopia [39] showed that STEC strains isolated from raw milk samples harbored the highest prevalence of resistance against streptomycin (81.80%), trimethoprim-sulfamethoxazole (27.30%), cefoxitin (54.50%), kanamycin (63.60%), gentamicin (36.40%), tetracycline (81.80%) and norfloxacin (51.50%) which was similar to our findings. An Italian investigation [22] reported that STEC strains isolated from dairy products exhibited the highest levels of resistance against ampicillin, gentamicin, tetracycline, sulfamethoxazole and ciprofloxacin antibiotics.

The most significant finding obtained from our study and also those of other researches were the high prevalence of resistance against human-based antibiotics such as amikacin, imipenem, cefotaxime, ciprofloxacin, cotrimoxazole and ceftazidime. This finding showed that STEC strains isolated from raw milk and traditional dairy product samples may be transferred from infected humans. Therefore, some of the STEC strains of our study may be had anthropogenic origin and derived from infected staffs of the milking halls and also dairy producing factories. This finding was also supported by previous studies [5–7, 29, 31, 34].

We also found a considerable prevalence of resistance of STEC strains against chloramphenicol (4.68%). However, chloramphenicol was listed as a forbidden antibiotic, but its highly irregular and excessive prescription may be cause its high prevalence of resistance. High prevalence of resistance against chloramphenicol was also reported by other studies [5–7, 17, 18, 29, 31, 40]. Findings of other investigations revealed that the STEC strains isolated from food samples harbored the high prevalence of resistance against chloramphenicol (1 to 50%) [5–7, 17, 18, 29, 31, 40]. Differences in the prevalence of resistance against various types of antibiotics observed in our study and also other researches are may be due to the differences in the availability of antibiotics, their costs and finally idea of veterinarian for antibiotic prescription in various parts of the world.

High presence of virulent and resistant *E. coli* and also other foodborne pathogens have been reported from different types of food samples [41–58]. Findings of these researchers and also those of our investigation showed that pathogenic bacteria such as *E. coli* are not limited to health-care centers. In the other hand, community and especially foods may play an important role in transmission of pathogenic bacteria such as *E. coli* to humans. Furthermore, they are mainly act as reservoir of pathogenic bacteria such as *E. coli* in the community. Raw milk and traditional dairy product samples are not exception from this important regulation. Therefore, further studies are needed to determine the role of different types of food samples as risk factor for survival and transmission of pathogenic bacteria such as *E. coli* in the community. In addition to food, presence of *E. coli* strains have been reported previously from different types of clinical infections [59–74].

## Conclusions

In conclusions, we recognized a considerable prevalence of O26 and O157 serogroups, *stx1* and *eaeA* virulence factors, resistance against ampicillin, gentamicin and tetracycline and *aac(3)-IV*, *CITM* and *tetA* antibiotic resistance genes in the STEC strains isolated from raw milk and traditional dairy products. These characters besides the high prevalence of multi-drug resistant STEC strains represented an important public health issue regarding the consumption of raw milk and traditional dairy products. High prevalence of O157 serogroup which was accompanied with simultaneous presence of two or more putative virulence factors together showed their high pathogenicity. We found that kashk and dough were safe from contamination with *E. coli* strains. In addition, the prevalence of *E. coli* strains in raw camel milk samples was only 6.6%. Presence of O157 serogroups, EHEC strains, human-based antibiotics and even antibiotic resistance genes which encode resistance against human-based antibiotics in dairy products showed insufficiency of cooking time and temperature and transmission of pathogenic bacteria from infected staffs into the dairy samples. It seems that there were no strict supervisions on the principles of food hygiene in Iranian factories. Due to the low prevalence of STEC resistance against imipenem antibiotic, occurrence of food poisonings due to the STEC strains in tested samples can be treated with its regular prescription. Attentions to the principles of Hazard Analysis and Critical Control Point (HACCP) system can reduce the risk of STEC strains in raw milk and traditional dairy products. Complete boiling of raw milk before consumption and using from pasteurized dairy products were recommended to decrease the transmission of STEC strains to human. Attentions to the results of disk diffusion and principles of antibiotic prescription can decrease the risk of resistant STEC strains in raw milk and traditional dairy products.

## Abbreviations

AEEC: Attaching and effacing *E. coli*
*E. coli*: *Escherichia coli*
*Eae*: Intimin
EHEC: Enterohemolysin or Enterohaemorrhagic *Escherichia coli*
*Ehly*: Hemolysin
HC: Hemorrhagic colitis
HUS: Hemolytic Uremic Syndrome
PCR: Polymerase Chain Reaction
SPSS: Statistical Package for the Social Sciences
STEC: Shiga toxin producing *Escherichia coli*.
*Stx*: Shiga toxin
